# Evaluation of Rapid Testing Algorithms for Venue-based Anonymous HIV Testing among Non-HIV-Positive Men Who Have Sex with Men, National HIV Behavioral Surveillance (NHBS), 2017

**DOI:** 10.1007/s10900-020-00871-3

**Published:** 2020-07-10

**Authors:** Shamaya Whitby, Amanda Smith, Rebecca Rossetti, Johanna Chapin-Bardales, Amy Martin, Cyprian Wejnert, Silvina Masciotra, for the NHBS Study Group, Pascale Wortley, Jeff Todd, David Melton, Monina Klevens, Rose Doherty, Conall O’Cleirigh, Stephanie Masiello Schuette, Antonio D. Jimenez, Jonathon Poe, Margaret Vaaler, Jie Deng, Alia Al-Tayyib, Melanie Mattson, Vivian Griffin, Emily Higgins, Mary-Grace Brandt, Salma Khuwaja, Zaida Lopez, Paige Padgett, Ekow Kwa Sey, Yingbo Ma, Emma Spencer, Willie Nixon, David Forrest, Bridget Anderson, Ashley Tate, Meaghan Abrego, William T. Robinson, Narquis Barak, Jeremy M. Beckford, Sarah Braunstein, Alexis Rivera, Sidney Carrillo, Barbara Bolden, Afework Wogayehu, Henry Godette, Kathleen A. Brady, Chrysanthus Nnumolu, Jennifer Shinefeld, Sean Schafer, E. Roberto Orellana, Amisha Bhattari, H. Fisher Raymond, Theresa Ick, Sandra Miranda De León, Yadira Rolón-Colón, Tom Jaenicke, Sara Glick, Celestine Buyu, Toyah Reid, Karen Diepstra, Monica Adams, Christine Agnew Brune, Qian An, Alexandra Balaji, Dita Broz, Janet Burnett, Johanna Chapin-Bardales, Melissa Cribbin, YenTyng Chen, Paul Denning, Katherine Doyle, Teresa Finlayson, Senad Handanagic, Brooke Hoots, Wade Ivy, Kathryn Lee, Rashunda Lewis, Evelyn Olansky, Gabriela Paz-Bailey, Taylor Robbins, Catlainn Sionean, Amanda Smith, Cyprian Wejnert, Mingjing Xia

**Affiliations:** 1 Oak Ridge Institute for Science and Education at the Division of HIV/AIDS Prevention, National Center for HIV/AIDS, Viral Hepatitis, Division of HIV/AIDS Prevention, National Center for HIV/AIDS, Viral Hepatitis, STD and TB Prevention, Centers for Disease Control and Prevention, 1600 Clifton Rd NE MS-A25, Atlanta, GA 30329 USA; 2 Division of HIV/AIDS Prevention, National Center for HIV/AIDS, Viral Hepatitis, STD and TB Prevention, Centers for Disease Control and Prevention, 1600 Clifton Rd NE, Atlanta, GA 3033 USA

**Keywords:** HIV rapid testing, HIV rapid tests algorithm, False-reactivity, Discordant RT result

## Abstract

HIV rapid testing algorithms (RTAs) using any two orthogonal rapid tests (RTs) allow for on-site confirmation of infection. RTs vary in performance characteristics therefore the selection of RTs in an algorithm may affect identification of infection, particularly if acute. National HIV Behavioral Surveillance (NHBS) assessed RTAs among men who have sex with men recruited using anonymous venue-based sampling. Different algorithms were evaluated among participants who self-reported never having received a positive HIV test result prior to the interview. NHBS project areas performed sequential or parallel RTs using whole blood. Participants with at least one reactive RT were offered anonymous linkage to care and provided a dried blood spot (DBS) for testing at CDC. Discordant results (RT-1 reactive/RT-2 non-reactive) were tested at CDC with lab protocols modified for DBS. DBS were also tested for HIV-1 RNA (VL) and antiretroviral (ARV) drug levels. Of 6500 RTAs, 238 were RT-1 reactive; of those, 97.1% (231/238) had concordant results (RT-1/RT-2 reactive) and 2.9% (7/238) had discordant results. Five DBS associated with discordant results were available for confirmation at CDC. Four had non-reactive confirmatory test results that implied RT-1 false reactivity; one had ambiguous confirmatory test results which was non-reactive in further testing. Regardless of order and type of RT used, RTAs demonstrated high concordant results in the population surveyed. Additional laboratory testing on DBS following discordant results confirmed no infection. Implementing RTAs in the context of anonymous venue-based HIV testing could be an option when laboratory follow-up is not practicable.

## Introduction

Nonclinical testing settings allow HIV testing programs to reach populations at increased risk who otherwise might not engage in regular HIV testing. Confirmation of HIV infection in nonclinical settings can expedite linkage to care for newly infected persons, those unaware of infection, and persons previously diagnosed but not in care [1, 2]. The 2016 guidelines published for point-of-care (POC) HIV testing in nonclinical settings highlighted three possible testing algorithms: (1) a single rapid test with immediate linkage to clinical provider if initial test is reactive, (2) a single rapid test followed by laboratory-based follow-up testing if initial test is reactive, or (3) a rapid test immediately followed by a second rapid test on-site if the initial test is reactive [2–4]. We evaluated the third nonclinical testing algorithm within National HIV Behavioral Surveillance (NHBS), which conducts anonymous cross-sectional behavioral surveys and HIV testing in annual rotating cycles with men who have sex with men (MSM), persons who inject drugs, and heterosexually active persons at increased risk for HIV. In 2017, NHBS recruited a large sample of MSM using venue-based sampling in 23 U.S. metropolitan statistical areas [5, 6].

MSM are a population at increased risk for HIV acquisition who could benefit from access to low-barrier HIV testing. In the 2014 NHBS MSM survey, one in five MSM were HIV positive and one in four who were positive were potentially unaware of their infection [5]. Until 2017, NHBS participants received one RT and, if reactive or preliminary positive, confirmation was performed using laboratory-based HIV IgG/IgM or Ag/Ab immunoassays at local or CDC laboratories. This created a barrier to knowledge of confirmed HIV status as participants needed to call or return in 1–2 weeks for confirmatory results [7]. In the U.S., an estimated 25–48% of participants who receive conventional HIV laboratory testing do not return for final confirmatory test results [8, 9]. A meta-analysis found that participants who received a RT were 1.5–2.2-times more likely to obtain their test results than those who received standard laboratory testing [10].

As indicated in 2016 guidelines for non-clinical settings, a rapid-rapid testing algorithm (RTA) allows participants to receive their results the same day that testing is performed. In NHBS, the use of a RTA was implemented in 2017 to replace laboratory confirmatory testing [7, 11]. Although the NHBS recommendation was to include two orthogonal RTs, the order (sequential or parallel) in which each RT was performed was not specified. For this analysis, the first RT performed in an RTA we define as RT-1 and the second as RT-2. If RTs were performed in parallel, RT-1 refers to the first RT conducted. Given that false-positive results are possible, employing an RTA allows for establishing concordance between the two tests (RT-1 reactive/RT-2 reactive) or whether they were discordant (RT-1 reactive/RT-2 non-reactive). Laboratory testing was included to address RT false-reactivity. This analysis evaluated the ability of different RTAs in non-clinical settings to provide confirmation of HIV infection status by calculating the frequency of concordant results and false-reactive results.

## Methods

### NHBS Survey

During 2017, MSM were recruited for NHBS using venue-based, time–space sampling at places such as clubs, bars, and street locations that are frequented by MSM [12]. Project areas used an anonymous standardized questionnaire to collect information on HIV-related risk behavior, the use of prevention services, and HIV testing, following national protocols with adaptations for study populations and local policies [13, 14]. Voluntary anonymous HIV testing was offered with counseling and anonymous linkage to care services. Based on their self-reported HIV status during the survey, participants were classified as either HIV-positive or non-HIV-positive. Self-reported non-HIV-positive (SRNH +) participants were defined as reporting never having received a positive HIV test result. This included participants who never received an HIV test or received an HIV test but had a negative or unknown test result or never received the test result.

### NHBS HIV Testing and Data Collection

NHBS project areas could have implemented a sequential RTA (s-RTA) involving two different orthogonal RTs conducted in sequence or a parallel RTA (p-RTA) in which two orthogonal RTs were conducted simultaneously. Orthogonal tests were either a similar kind of test but from two different manufacturers or two tests from the same manufacturer that relied on different principles of detection [6, 15]. NHBS project areas chose the RTs they used and the test order in their adopted algorithms; the tests used were any combination of INSTI® HIV-1/HIV-2 Antibody Test (INSTI; BioLytical, Richmond, BC), Alere Determine™ HIV–1/2 Ag/Ab Combo (DC; Alere Scarborough, Scarborough, ME), Uni-Gold™ Recombigen® HIV-1/2 (Unigold; Trinity Biotech, Bray, Ireland), Chembio SURE CHECK® HIV 1/2 Assay (Sure Check; Chembio Diagnostic Systems, Inc, Medford, NY), and OraQuick ADVANCE® Rapid HIV-1/2 Antibody Test (OraQuick; OraSure Technologies, Bethlehem, Pennsylvania). Eighteen project areas followed a s-RTA, one project area performed a p-RTA, and four project areas chose to use one RT followed by laboratory confirmation. Local laboratory confirmatory HIV testing was done using Avioq HIV-1 Microelisa System (Avioq, Inc., Rockville, MD) and/or HIV-1 Western Blot (WB; OraSure Technologies, Bethlehem, Pennsylvania: Bio-Rad Laboratories, Hercules, California) with DBS specimens or Abbott ARCHITECT HIV Ag/Ab Combo (Abbott Laboratories, Wiesbaden, Germany) with plasma specimens. Confirmation at CDC lab was performed using GS HIV Combo Ag/Ab EIA (BRC; Bio-Rad Laboratories) and Geenius HIV-1/2 supplemental assay (Geenius; Bio-Rad Laboratories) with DBS specimens. In project areas that implemented a s-RTA, all participants received a RT-1 and, if reactive, a RT-2 was performed. If the participant had any reactive RT and consented to blood storage, a DBS card was made from either finger-stick or venous whole blood for storage and future testing at CDC. In project areas that implemented a p-RTA, all participants received both RT-1 and RT-2 concurrently and DBS was also collected from participants that had any reactive RT and consented to blood storage. DBS were collected following a standard protocol, dried for a minimum of 4 h and up to 24 h at ambient temperature, and then placed in sealable bags with desiccants and a humidity indicator card [16]. The bagged DBS were stored in a dry environment at ambient temperature for up to a week before shipping to CDC. Once received DBS were inventoried and individually re-bagged with desiccants and a humidity indicator card and then stored at − 20 °C [17].

### Testing at CDC

DBS samples from participants with discordant results (RT-1 reactive/RT-2 non-reactive) were tested at the CDC lab to confirm HIV infection status. CDC used previously published protocols for GS HIV Combo Ag/Ab EIA (BRC; Bio-Rad Laboratories) and Geenius HIV-1/2 supplemental assay (Geenius; Bio-Rad Laboratories) which were validated for use with DBS in the CDC laboratory [18]. All samples received viral load (VL) testing using a modified protocol (HIV-1 RNA 1.0 mL extraction protocol) on the Abbott *Real*Time m2000sp HIV-1 VL kit (Abbott Molecular Inc., Des Plaines, IL) optimized for 70 µL of whole blood [19, 20]. Because DBS collected during the study may have limited quantity of blood, four 6 mm punches (~ 50 µL of whole blood) were used in a laboratory-validated protocol [21]. Blood was eluted in 1.3 mL of mLysis buffer in master mix tubes, both provided with the Abbott m Sample Preparation System (m2000sp). Samples were incubated at 55 °C for 30 min, vortexed, spun, and placed on m2000 for sample extraction and VL quantification according to the standard HIV-1 RNA quantitative assay 1.0 mL protocol [22].

The CDC lab also quantified concentrations of the antiretroviral drugs, Raltegravir (RAL), Tenofovir (TFV), Abacavir (ABC), Ritonavir (RTV), Lamivudine (3TC), Efavirenz (EFV), Emtricitabine (FTC), Elvitegravir (EVG) and Dolutegravir (DTG) in DBS using high-performance liquid chromatography-tandem mass spectrometry (HPLC–MS) (Sciex, Foster City, CA, Shimadzu Scientific, Columbia, MD). Briefly, 500 µL of 75% acetonitrile containing internal standards, isotopically labeled RAL, TFV, EVG and FTC (Toronto Research Chemicals Inc, Toronto, Canada), were added to one 6 mm punch and sonicated for 30 min to extract drugs. The supernatant was moved to a 96-well plate, evaporated to near dryness and re-suspended in 150 µL of mobile phase A (0.2% formic acid in water). Ten µL of the final solution was injected onto a UK-C18 column (100 × 1 mm, Imtakt, Portland, OR) connected to an HPLC–MS/MS system [23]. An aqueous-acetonitrile mobile-phase gradient was used to elute the drugs from the column and into the analyzer. Two mass transitions (Q1⟶Q3) (where possible) were monitored in positive MRM (Multiple Reaction Monitoring) mode which were as follows: RAL- 445.00 m/z⟶109.10 m/z and 445.00 m/z⟶361.20 m/z; TFV-288.00 m/z⟶176.30 m/z and 288.00 m/z⟶159.10 m/z; ABC-287.20 m/z⟶153.10 m/z and 287.20 m/z⟶191.00 m/z; 3TC-230.00 m/z⟶111.90 m/z; EFV- 316.10 m/z⟶244.20 m/z and 316.10 m/z⟶168.00 m/z; FTC-248.00 m/z⟶130.10 m/z and 248.00 m/z⟶113.10 m/z; RTV-721.20 m/z⟶296.20 m/z and 721.20 m/z⟶268.10 m/z; EVG-448.20 m/z⟶344.10 m/z and 448.20 m/z⟶143.10 m/z; and DTG-420.30 m/z⟶277.20 m/z and 420.30 m/z⟶127.10 m/z, respectively. The drug concentrations were estimated from a standard curve with a range of 0.5–2000 ng/mL constructed using spiked blood to make DBS of appropriate concentrations [24] and analyzed using Analyst software (Sciex, Foster City, CA). The lower limit of quantification (LOQ) for all analytes in this assay was between 10 and 25 ng/mL. The detectable concentrations were measured and reported in the ranges > 500 ng/mL (+ + +), 51–500 ng/mL (+ +), LOQ-50 ng/mL ( +) with the approximate days since last dose.

### SAS Analysis

This analysis was limited to non-HIV-positive participants in the 19 NHBS project areas that performed an s-RTA or p-RTA. The four project areas that performed one RT followed by local confirmation were excluded. Participants were eligible if they consented to the survey and provided valid, complete responses, consented to HIV testing, completed the s-RTA or p-RTA, had valid test results, and self-reported being non-HIV-positive. Only participants who further consented to DBS storage were included in analyses of CDC laboratory test results. Numbers and frequencies of rapid HIV test results by RTAs were calculated using SAS 9.4 (SAS Institute, Cary, NC).

## Results

During the 2017 MSM data collection, 11,232 participants were screened to participate in the 19 (18 s-RTA project areas and 1 p-RTA project area) NHBS project areas included in this analysis. Of the 6500 eligible participants, 6182 received a s-RTA while 318 received a p-RTA. Of 6182 receiving a s-RTA, the frequency at which each test was used for RT-1 was 2825 (45.7%) for DC; 1955 (31.6%) for INSTI; 528 (8.5%) for Unigold; and 874 (14.1%) for Sure Check. Regardless of the RT used 5960 (96.4%) were RT-1 non-reactive and received no further testing; 222 (3.6%) were RT-1 reactive and received a RT-2. Of the 222 RT-1 reactive, 216 (97.3%) were RT-2 reactive (concordant results) and 6 (2.7%) were RT-2 non-reactive (discordant results).

Tables 1 through 3 show the results of specific s-RTAs adopted in 18 project areas with concordance and discordance reported for each combination of RTs. Of 2825 participants testing initially with DC, 2295 (81.2%) were tested with INSTI as a second RT, 404 (14.3%) with Sure Check and 126 (4.5%) with Unigold. There was an overall concordance of 110/115 (95.7%) when DC was followed by any of the RTs while 5.2% participants tested with DC followed only by INSTI had discordant results (Table 1).

**Table 1:** Variation of sequential RTAs that started with Determine Combo among non-HIV-positive men who have sex with men in 19 U.S. cities—National HIV Behavioral Surveillance, 2017

Rapid testing algorithm (RTA)	Number of participants tested according to RTA	RT-1-Non-reactive % (fraction)	Concordant RT (%)^1^	Discordant RT (%)^2^
			RT-1-R- > RT-2-R % (fraction)	RT-1-R- > RT-2-NR % (fraction)
DC→INSTI	2295	95.8 (2198/2295)	94.8 (92/97)	5.2 (5/97)
DC→Sure Check	404	96.3 (389/404)	100 (15/15)	0
DC→Unigold	126	97.6 (123/126)	100 (3/3)	0

HIV rapid tests: DC: Alere Determine HIV-1/2 Ag/Ab Combo Rapid Test, INSTI: BioLytical INSTI HIV -1/ HIV-2 Antibody Test, Sure Check: Chembio SureCheck HIV-1/2 Assay, Unigold: Trinity Biotech Uni-gold Recombigen HIV-1/2

*N* Number of nonreactive or reactive rapid test results

(%)^1^ represents the concordance percentage for each of the combinations of RTs and (%)^2^ represents the discordant percentage for each of the combinations of RTs

For the s-RTA starting with INSTI, 1955 participants were initially tested of which 1134 (58%) participants were tested with DC as a second RT, 469 (24%) with Sure Check, and 352 (18%) with Unigold. There was an overall concordance of 46/47 (97.9%) when INSTI was followed by DC, Sure Check, or Unigold. While 3.3% of participants tested using INSTI followed by DC had discordant results (Table 2).

**Table 2:** Variation of sequential RTAs that started with INSTI among non-HIV-positive men who have sex with men in 19 U.S. cities—National HIV Behavioral Surveillance, 2017

Rapid testing algorithm (RTA)	Number of participants tested according to RTA	RT-1-Non-reactive % (fraction)	Concordant RT (%)^1^	Discordant RT (%)^2^
			RT-1-R- > RT-2-R % (fraction)	RT-1-R- > RT-2-NR % (fraction)
INSTI→DC	1134	97.3 (1104/1134)	96.7 (29/30)	3.3 (1/30)
INSTI→Sure Check	469	96.6 (453/469)	100 (16/16)	0
INSTI→Unigold	352	99.7 (351/352)	100 (1/1)	0

HIV rapid tests: INSTI: BioLytical INSTI HIV -1/ HIV-2 Antibody Test, DC: Alere Determine HIV-1/2 Ag/Ab Combo Rapid Test, Sure Check: Chembio SureCheck HIV-1/2 Assay, Unigold: Trinity Biotech Uni-gold Recombigen HIV-1/2.

*N* Number of nonreactive or reactive rapid test results

(%)^1^ represents the concordance percentage for each of the combinations of RTs and (%)^2^ represents the discordant percentage for each of the combinations of RTs

Several project areas performed other variations of a s-RTA (Table 3). Only one s-RTA started with Unigold and was followed by INSTI which 528 (100%) participants were tested. For the s-RTA starting with Sure Check 874 participants were initially tested. Using INTSI as the second RT 851 (97.4%) participants were tested and 23 (2.6%) were tested with OQ. Unigold followed by INSTI and Sure Check followed by INSTI or OQ showed 100% concordance.

**Table 3:** Sequential RTAs that started with Unigold or Sure Check among non-HIV-positive men who have sex with men in 19 U.S. cities—National HIV Behavioral Surveillance, 2017

Rapid testing algorithm (RTA)	Number of participants tested according to RTA	RT-1-Non-Reactive % (fraction)	Concordant RT (%)^1^	Discordant RT (%)^2^
			RT-1-R- > RT-2-R % (fraction)	RT-1-R- > RT-2-NR % (fraction)
Unigold→INSTI	528	94.3 (498/528)	100 (30/30)	0
SureCheck→INSTI	851	96.7 (823/851)	100 (28/28)	0
SureCheck→OQ	23	91.3 (21/23)	100 (2/2)	0

Sure Check: Chembio SureCheck HIV-1/2 Assay, INSTI: BioLytical INSTI HIV -1/ HIV-2 Antibody Test, Unigold: Trinity Biotech Uni-gold Recombigen HIV-1/2, OQ: OraQuick ADVANCE Rapid HIV-1/2 Antibody Test

*N* Number of nonreactive or reactive rapid test results

(%)^1^ represents the concordance percentage for each of the combinations of RTs and (%)^2^ represents the discordant percentage for each of the combinations of RTs

A p-RTA was adopted in one project area which screened 318 participants using Sure Check and OraQuick. In the p-RTA, participants received two orthogonal RT concurrently which returned 16 RT reactive on the first RT used. Fifteen (4.7%) participants had concordant reactive results and 302 (95%) participants had concordant nonreactive results. One (0.3%) participant had discordant results (SureCheck reactive, OraQuick non-reactive). The overall concordance for this algorithm was 99.6%.

Table 4 shows the testing performed to confirm HIV infection in samples from participants with discordant results in s-RTAs/p-RTAs. Although DC can differentiate Ag/Ab reactivity, the individual analyte reactivity data was not collected; only the overall reactive or non-reactive results were recorded. Of the five participants (samples 1–5) with discordant results in the DC-INSTI s-RTA, three consented to DBS storage and had DBS samples sent to CDC for confirmatory testing. The BRC was nonreactive for these and, therefore, Geenius was not performed following the recommended HIV diagnostic algorithm. Undetectable VL results and ARVs in samples 1 and 2 suggested DC false reactivity. Sample 3 had ~ 50 ng/mL of Dolutegravir (DTG) which could not exclude viral suppression. Sample 4 was HIV-1 WB antibody-negative which did not confirm the DC Ag/Ab-reactive result based on local testing, a DBS sample was not sent to CDC. Sample 5 did not receive confirmatory testing locally or at CDC lab as a sample was not received. The s-RTA starting with INSTI followed by DC had one participant with discordant results (sample 6); this sample was BRC reactive but Geenius HIV-1 Ab negative and VL and drug levels were also not detected, likely suggesting false reactivity in both the INSTI and BRC screening tests. In the p-RTA with Sure Check and OraQuick, one participant had discordant results (sample 7). This sample was tested locally and at CDC where WB, BRC, and VL results indicated Sure Check false reactivity.

**Table 4:** Results of confirmatory testing on dried blood spot specimens from non-HIV-positive men who have sex with men with discordant rapid test results—National HIV Behavioral Surveillance, 19 U.S. cities, 2017

Sample number	RT/results 1	RT/results 2	Comments/local testing	Bio-Rad GS Ag/Ab Combo EIA (BRC)	Bio-Rad Geenius HIV-1/2 Supplemental	Abbott *Real*Time HIV-1 RNA assay (copies/mL)	Antiretroviral drugs
1	DC-R	INSTI-NR		Non-Reactive		Target not detected	Not detected
2	DC-R	INSTI-NR		Non-Reactive		Target not detected	Not detected
3	DC-R	INSTI-NR		Non-Reactive		Target not detected	DTG (+ /4)
4	DC-R	INSTI-NR	WB HIV-negative DBS not sent				Not done
5	DC-R	INSTI-NR	DBS not sent				Not done
6	INSTI-R	DC-NR		Reactive (S/CO = 10.1)	HIV-1 Antibody Negative	Target not detected	Not detected
7	Sure Check-R	OQ-NR	WB HIV-negative	Non-Reactive		Target not detected	Insufficient quantity

*DTG* Dolutegravir (Tivicay) at ~ 50 ng/ml; *R* reactive; *NR* non-reactive; *WB* Western Blot; *S/CO* signal cut-off is calculated by dividing the OD value by assay cut off value

## Discussion

Using two orthogonal RTs to confirm HIV infection in non-clinical settings can allow for same day return of results and expedited linkage to care. We demonstrate that the high concordance of RT results in using an RTA supports that testing with two RTs is a viable option for diagnosis in populations at increased risk when conventional laboratory testing settings might not be readily accessible. Most discordant results could be attributed to RT-1 false reactivity in five of the seven cases.

While false-reactivity in HIV RTs has been widely reported, usually leading to discordant RT results, it is not common [25]. False-reactive results can occur for many reasons including but not limited to technical issues with the test device, mislabeling, improper handling, or misinterpretation of a visually read test [26]. The s-RTA starting with DC followed by INSTI had 5% (5/97) discordant results, INSTI followed by DC had 3.3% (1/30) discordant results, and Sure Check/OraQuick in the p-RTA had 0.3% (1/318) discordant results. Follow-up laboratory results suggest false-reactivity in few of the discordant samples, although results were inconclusive for others. False- reactivity was confirmed by further laboratory testing in DC followed by INSTI s-RTA (samples 1–4), although the source of the detected ARV in sample 3 remains unknown and could for instance be related to post-exposure prophylaxis. In the p-RTA, Sure Check/OraQuick (sample 7) indicate false reactivity in both screening tests after laboratory confirmation via WB was performed for one sample. VL and drug levels did not confirm reactivity in INSTI and BRC (sample 6). However, we cannot discount that low viremia and ARV drug exposure could have been missed by the assays or that the participant was an elite controller (suppressed HIV-1 RNA levels to below the limit of detection in the absence of ART) [27]. In contrast, if it were an acute infection, HIV-1 RNA levels would have been higher than the limit of quantification of our validated VL assay. In the p-RTA, Sure Check/OraQuick indicate false reactivity in both screening tests after laboratory confirmation via WB was performed for one sample.

Confirmatory testing limitations include the use of WB for confirmation after screening with DC since WB cannot detect p24 antigen. Moreover, the WB assay faces many challenges such as high rate of uncertainty, low sensitivity, long testing period, and the limitation of detection in the early stages of seroconversion, at a time when cost of the assay is increasing [28–30]. During this cycle, we did not collect the individual analyte reactivity to address the impact of Ag false-reactivity frequently reported from field studies [31]. Other FDA-approved supplemental tests are available for validated off-label use with DBS or laboratory-developed tests could be used. Another confirmatory testing limitation is the sensitivity of the lab-validated Abbott *Real*Time HIV-1 assay using four DBS punches (~ 50 µL), instead of 70 µL whole blood, that detects 66% of 3 log (copies/mL) [21]; thus participants with low viremia may have been missed.

In following the s-RTA as designed, participants who self-reported being non-HIV-positive and had a non-reactive RT-1 result only received one RT; these participants were considered HIV-negative and did not receive a second RT. These samples were not sent to CDC for testing to evaluate whether any acute infections may have been missed. While RT kits are more expensive and less sensitive than conventional blood test on serum/plasma, research suggests that RTs are more cost-effective when factoring the number of people tested who receive their results when compared to conventional testing [32, 33]. A randomized trial at an STI clinic found that 88% of MSM preferred RT to standard blood testing and a systematic review showed that 87–97% of clients would choose a RT over standard blood testing [7, 34]. It is estimated that desirability of RTs would increase uptake of HIV testing three-fold compared to standard blood testing in community-based voluntary counseling and testing settings or when approached by HIV counselors in emergency departments [7, 35–37]. Those who self-reported being HIV-positive with a negative RT result received confirmatory testing at CDC; these findings are being analyzed separately.

## Conclusion

In 2016, approximately 40% of people who were unaware of their HIV infection status or who had been previously diagnosed but not engaged in care or treatment contributed to 80% of new HIV infections in the US [38]. Using an RTA to provide definitive results may expedite linkage to care in populations at increased risk who otherwise might not seek testing. The high concordance of RTs in both the s-RTA and p-RTA sites has shown that implementing an algorithm for testing high-risk populations in non-clinical settings effectively returns results to participants who may experience barriers to receiving confirmatory results from a laboratory. The few participants with discordant results, most confirming as HIV-negative, suggested that a two-test RTA provides a reliable indication of infection status, notwithstanding persons with acute infection. The results did not find that any particular RTA performed better than another as the sample size of each algorithm differed limiting comparisons between the RTAs. Before choosing or implementing an RTA for a setting, the characteristics and HIV prevalence of the target population, characteristics and costs of RTs, and access to lab-based testing should be considered. RTAs will continue to be analyzed in the upcoming cycles of NHBS to evaluate effectiveness of different RTAs used.
